# O-Cyclic Phytosphingosine-1-Phosphate Protects against Motor Dysfunctions and Glial Cell Mediated Neuroinflammation in the Parkinson’s Disease Mouse Models

**DOI:** 10.3390/antiox11112107

**Published:** 2022-10-26

**Authors:** Hyeon Jin Lee, Kyonghwan Choe, Jun Sung Park, Amjad Khan, Min Woo Kim, Tae Ju Park, Myeong Ok Kim

**Affiliations:** 1 Division of Life Science and Applied Life Science (BK21 FOUR), College of Natural Sciences, Gyeongsang National University, Jinju 52828, Korea; 2 Department of Psychiatry and Neuropsychology, School for Mental Health and Neuroscience (MHeNs), Maastricht University, 6229ER Maastricht, The Netherlands; 3 Haemato-oncology/Systems Medicine Group, Paul O’Gorman Leukaemia Research Centre, Institute of Cancer Sciences, College of Medical, Veterinary & Life Sciences (MVLS), University of Glasgow, Glasgow G12 0ZD, UK; 4 Alz-Dementia Korea Co., Jinju 52828, Korea

**Keywords:** Parkinson’s disease, O-cyclic phytosphingosine-1-phosphate, motor dysfunction, α-synuclein, dopaminergic neuron, oxidative stress, glial cell, neuroinflammation, neuroprotection

## Abstract

O-cyclic phytosphingosine-1-phosphate (cPS1P) is a novel and chemically synthesized sphingosine metabolite derived from phytosphingosine-1-phosphate (S1P). This study was undertaken to unveil the potential neuroprotective effects of cPS1P on two different mouse models of Parkinson’s disease (PD). The study used 1-methyl-4-phenyl-1,2,3,6-tetrahydropyridine (MPTP) and neuron specific enolase promoter human alpha-synuclein (NSE-hαSyn) Korl transgenic mice. MPTP was injected for five consecutive days and cPS1P was injected for alternate days for six weeks intraperitoneally. We performed behavioral tests and analyzed the immunohistochemistry and immunofluorescence staining in the substantia nigra pars compacta (SNpc) and the striatum. The behavior tests showed a significant reduction in the motor functions in the PD models, which was reversed with the administration of cPS1P. In addition, both PD-models showed reduced expression of the sphingosine-1-phosphate receptor 1 (S1PR1), and α-Syn which was restored with cPS1P treatment. In addition, administration of cPS1P restored dopamine-related proteins such as tyrosine hydroxylase (TH), vesicular monoamine transporter 2 (VMAT2), and dopamine transporter (DAT). Lastly, neuroinflammatory related markers such as glial fibrillary acidic protein (GFAP), ionized calcium-binding adapter protein-1 (Iba-1), c-Jun N-terminal kinases (JNK), nuclear factor kappa-light-chain-enhancer of activated B cells (NF-kB), tumor necrosis factor-alpha (TNF-α), and interleukin 1 beta (IL-1β) were all reduced after cPS1P administration. The overall findings supported the notion that cPS1P protects against dopamine depletion, neuroinflammation, and PD-associated symptoms.

## 1. Introduction

Parkinson’s disease (PD) is the second most common neurodegenerative disease, affecting 1–2% of the population older than 65 years. It is a progressive neurodegenerative disease and clinically characterized by resting tremor, rigidity, bradykinesia, slurred speech, postural instability, and cognitive impairment [1,2,3]. The pathological hallmarks of PD include degeneration of nigrostriatal dopaminergic neurons and the presence of neuritic and cytoplasmic deposition of α-synuclein (α-Syn) [4]. The combination of dopaminergic neurodegeneration and accumulation of α-Syn leads to dopamine depletion in the striatum, causing motor and other clinical symptoms of the disease [5]. In addition, neuroinflammation in the brain is a prominent feature of PD pathogenesis.

One of several factors explored in the pathophysiology of PD is sphingolipid metabolite sphingosine-1-phosphate (S1P) [6,7,8]. S1P is a lipid second messenger formed from the phosphorylation of sphingosine-by-sphingosine kinase. Biological activities of S1P are mediated by its function as a ligand for five G protein-coupled S1P receptors (S1PR1–5) [9,10]. S1P modulates the S1P receptors to regulate cell migration, immune modulation, mitochondrial apoptosis, inflammation, and neuronal survival. Therefore, S1P receptors are expressed in several cell types such as immune cells, neurons, microglia, astrocytes, and oligodendrocytes [11,12,13]. In the central nervous system (CNS), S1P plays an important role in neuronal protection and regulation of synaptic transmission through membrane excitability and neurotransmitter release [14,15,16]. Several lines of studies have suggested reduced expression of S1PR1 in PD-like conditions, and it has been suggested that pharmacological agonists of S1PR1 are responsible for significant regulation of PD-associated neuroinflammation and neurodegeneration [7,17]. Furthermore, down-regulation of S1PR1 has shown activation of inflammatory mediators, apoptotic cell death, synaptic dysfunction, and neurodegeneration [18], and increased level of reactive oxygen species (ROS) [19]. Indeed, oxidative stress is involved in the cascade leading to the degeneration of dopaminergic cells in PD and is associated with degenerative processes such as mitochondrial dysfunction and inflammation [20]. A significant increase in ROS was also found in treatment with 1-methyl-4-phenyl-1,2,3,6-tetrahydropyridine (MPTP) and Rotenone, chemicals commonly used in PD studies [21,22,23].

Unfortunately, S1P has limitations on its physiochemical characteristic and, most importantly, it is challenging to extract and quantify [24]. O-cyclic phytosphingosine-1-phosphate (cPS1P) is a novel sphingosine metabolite derived from phytosphingosine-1-phosphate (P1P), a homolog of S1P. cPS1P was synthesized in the O-linked cyclic of P1P as monohydric 18-carbon amino alcohol like S1P. In a previous study, cPS1P regulated the mammalian target of rapamycin (mTOR)-dependent hypoxia-induced factor α-subunits (HIFα) translation and nuclear translocation as the therapeutic potential of mesenchymal stem cells [25]. Although its functions remain to be described, cPS1P provides new biological properties to P1P through changes in the specificity and binding affinity to S1PR1. Therefore, the present study investigated the effects of cPS1P in PD pathology using two different mouse models.

## 2. Materials and Methods

### 2.1. Cell Culture

Human neuroblastoma SH-SY5Y cells were purchased from Korea Cell Line Bank (KCLB, South Korea) and cultured in Dulbecco’s Modified Eagle Medium (DMEM; Gibco, NY, USA) containing 1% antibiotic-antimycotic solution and 10% fetal bovine serum incubated at 37 °C with 5% CO_2_. The EGFP-alpha-synuclein-A53T plasmid was a gift from David Rubinsztein (Addgene #40823). Cells were transfected using Lipofectamine 3000 (Invitrogen, CA, USA) according to the instructions provided by the manufacturer. The SH-SY5Y cells were plated in 96-well plate, then cells were treated with 1-methyl-4-phenylpyridinium (MPP+) (Sigma-Aldrich, MO, USA) and were incubated with different concentrations of cPS1P (1, 10, 50 µM) for 24 hours (h) at 37 °C. Cell viability (400_EX_/505_Em_) and cytotoxicity (485_Ex_/520_Em_) were measured by fluorometrically. Apoptosis was measured by luminogenic caspase 3/7 substrate according to the instructions provided by manufacturer.

### 2.2. Animals

For MPTP injections, male wild-type C57BL/6J mice (approximately 25–28 g, 8 weeks, n = 8 mice/group/model) were purchased from Samtako Biolabs (Ulsan, South Korea). α-Syn transgenic (Tg) mouse overexpressing human α-syn under the control of the neuron specific enolase (NSE) promoter (C57BL/6N-Tg [NSE-hαSyn] Korl; approximately 25–28g, 8 weeks, n = 8 mice/group/model) were gifted from the National Institute of Food and Drug Safety Evaluation (NIFDS, Cheongju, Korea). The animals were genotyped according to the polymerase chain reaction (PCR) protocols of NIFDS using samples obtained from mouse tails. The mice were acclimated for 1 week under a 12 h dark/light cycle at 21–23 °C with 60 ± 10% humidity and provided food and water ad libitum. MPTP (Sigma-Aldrich, MO, USA) was dissolved in distilled water and administered intraperitoneally (i.p.) to the adult mouse (30 mg/kg) for 5 consecutive days, as suggested previously [26]. cPS1P dose was determined by previous studies which used another form of S1P agonist, fingolimod (FTY720). These studies all used the concentration of 1mg/kg [17,27,28]. Therefore, for this study, cPS1P dose was set as 1mg/kg. cPS1P was provided by Axcesobiopharma (Anyang, Korea); it was dissolved in 0.09N NaOH solution and administered intraperitoneally (i.p.) to the adult mouse (1 mg/kg) for alternate days for 6 weeks. The wild type C57BL/6J mice were randomly divided into the following three groups: (1) wild-type (WT; vehicle-treated), (2) MPTP (MPTP-treated group), and (3) MPTP + cPS1P. The C57BL/6-Tg (NSE-hαSyn) Korl mice were divided into the following four groups: (1) wild-type (WT; vehicle-treated), (2) NSE-hαSyn (α-Syn Tg), (3) α-Syn Tg + cPS1P, and 4) control + cPS1P (cPS1P).

### 2.3. Behavioral Tests

#### 2.3.1. Open-Field Test

The apparatus used for the open field test consists of an open-field box (40 × 40 cm with a height of 40 cm), which was divided into 16 equal-sized squares. Each mouse was placed in the center of the arena and allowed to freely explore it. The movements of the mice were recorded with a SMART video tracking system (Panlab, MA, USA). The tests were conducted in a sound-proof room under low light to prevent distractions and unintentional freezing behaviors.

#### 2.3.2. Pole Test

The pole used for the test consists of a wooden stick (40 cm in length and 10 mm in diameter). Mice were acclimatized to the behavioral room and received 3 trainings per day for 2 consecutive days. The mouse was placed on the top of the vertical wooden stick with the head in the face-up position. The total time (T-LA) taken to arrive at the bottom of the pole and place the forefeet on the floor was noted.

#### 2.3.3. Wire Hang Test

The wire hang test apparatus consisted of an iron wire stretched between two pots horizontally 20cm above the ground. Each mouse was placed on the wire, held with its forelimbs, and the time that each mouse remained on the wire was recorded. The test was repeated 8 times for each group, with the mouse resting between trials.

#### 2.3.4. Rotarod Test

The rotarod test apparatus consists of four adjacent rods, which are 40cm in height and 10cm in width. Each mouse was placed on the rods, which rotated with increasing speed. Mice were trained 10, 15 and 20 revolutions per minute (rpm). The experiments were repeated three times (3 min each). The time that the mouse remained on the rod was recorded, and the meantime was used for analysis. 

### 2.4. Tissue Sample Preparation for Morphological Analysis

After treatment and behavioral analysis, the mice were perfused transcardially with normal saline (0.9%) and 4% paraformaldehyde as described previously [29]. The brain was fixed with paraformaldehyde for 72 h at 4oC and then kept in the sucrose phosphate buffer (20%) for 72 h. The brain was frozen in the Tissue-Tek^®^ optimal cutting temperature (O.C.T) compound (Sakura Finetek USA, Inc., Torrance, CA, USA), and the sections were taken by CM3050C cryostat (Leica Germany). The brain sections were thaw-mounted on gelatin-coated slides (Fisher, Rock-ford, IL, USA).

### 2.5. Protein Extraction and Western Blot Analysis

Protein extraction and western blot was conducted as previously described [30,31]. Briefly, proteins were extracted using Pro-Prep protein extraction solution (iNtRON Biotechnology, Sungnam, Korea), and the protein concentration was measured using a spectrometer and Bio-Rad Assay Kit (Bio-Rad Laboratories, CA, USA). Western blot (n = 5 mice/group/model) as follows: for western blot, proteins were separated by SDS-PAGE 4~12% (Bio-Rad Laboratories, CA, USA) and transferred to polyvinylidene-difluoride (PVDF) membrane. The membrane was blocked in 5% (*w*/*v*) skimmed milk (DifcoTM- Skim Milk, BD, MD, USA) and incubated overnight at 4 °C with respective primary antibody for 16 h (Table 1). After incubation with primary antibody, the membrane was reacted with secondary antibody and washed with phosphate-buffered saline—0.1% Tween 20 (PBS-T). The immune reactivity was detected on X-ray films using the enhanced chemiluminescent (ECL) detection reagent (EzWestLumiOne, Korea) as a fluorescent reagent. The films were scanned and measured by ImageJ software and presented graphically. 

### 2.6. Immunohistochemistry

Immunohistochemistry was performed as described in previous study [32]. Briefly, immunohistochemistry (n = 3 mice/group/model) was conducted as follows. The slides were washed with PBS and treated with 10% H_2_O_2_ in methanol for 10 min and proteinase K was applied for 10 min. After proteinase K, the slides were treated with a blocking solution containing 5% BSA, normal goat serum, and Triton X-100 for 90 min. After that, the slides were incubated with diluted primary antibody (1:100 ~ 1:200 in blocking solution) at 4 °C overnight for 16 h (Table 1). After overnight treatment with the respective primary antibody, the slides were washed with PBS and incubated with respective secondary antibody (1:100 in PBS) for 2 h at room temperature and treated with ABC reagent (Vector Laboratories, CA, USA) for 1hr at room temperature and washed (with PBS for 10mins). Finally, the slides were treated with 3,3-diaminobenzidine tetrahydrochloride hydrated (DAB) solution (Sigma-Aldrich, St. Louise, USA) containing H_2_O_2_, dehydrated with ethanol (50%→70%→95%→100%), and treated in xylene for 5 min. Finally, the slides were covered with a glass coverslip using DPX mounting medium (Sigma-Aldrich, St. Louise, USA). Images were captured with Axioskop2 plus microscope (Zeiss, Stuttgart, Germany).

### 2.7. Immunofluorescence Assays

Immunofluorescence (n = 3 mice/group/model) was conducted as described previously [33,34]. Briefly, the slides were washed with 1% PBS for 10 min and incubated with proteinase K for 5 min at room temperature. The slides were washed and incubated with blocking solution, containing 2% normal serum and 0.3% Triton X-100 for 1 hr. After that, the slides were incubated with primary antibody overnight at 4 OC, washed, and reacted with fluorescent secondary antibody (Alexa Fluor 488 or 594, Invitrogen, CA, USA) for 2 h (Table 1). After incubation with secondary antibody, the brain sections were incubated overnight again with another primary antibody. The brain sections were washed and reacted with fluorescent secondary antibody (Alexa Fluor 488 or 594, Invitrogen, CA, USA) for 2 h under the same condition. The slides were washed with PBS and treated with 4′,6-diamidino-2-phenylindole (DAPI) (Invitrogen, CA, USA) for 10 min. Finally, slides were covered with glass coverslips using a fluorescence mounting medium (Agilent Dako, CA, USA). The images were captured with a confocal laser-scanning microscope (FluoView FV1000 MPE, Olympus, Tokyo, Japan).

### 2.8. Antibodies

All antibodies used in this study are given in Table 1.

### 2.9. Nissl Staining/Cresyl Violet Staining

Nissl staining was performed as previous described [35,36]. Briefly, the slides were washed with 1% PBS stained with 0.1% cresyl violet reagent (Sigma-Aldrich, St. Louis, MI, USA) for 10 min. After staining, slides were washed with distilled water and dehydrated with ethanol (70%→100%). Slides were immersed in differentiation solution (90% ethanol + acetic acid) and treated with xylene for 5 min and covered with glass coverslips using DPX mounting medium (Sigma-Aldrich, St. Louise, MI, USA).

### 2.10. Statistical Analysis

All western blot bands, immunohistological, and immunofluorescence images were quantified via ImageJ software. In western blot assay, the β-actin was used as a loading control. The densities were expressed as the mean ± standard error of mean (SEM). For statistical analysis and designing of graphs, we used GraphPad Prism 6 using one-way analysis of variance (ANOVA) followed by two-tailed independent Student’s t-test. The data were represented as the means ± SEM of the three independent biological and reproducible experiments. *p*-value less than 0.05 was considered statistically significant.

## 3. Results

### 3.1. cPS1P Attenuates MPP+-Induced Cell Viability Reduction, Cytotoxicity, and Apoptosis in SH-SY5Y Human Neuroblastoma Cells

**Figure 1:**
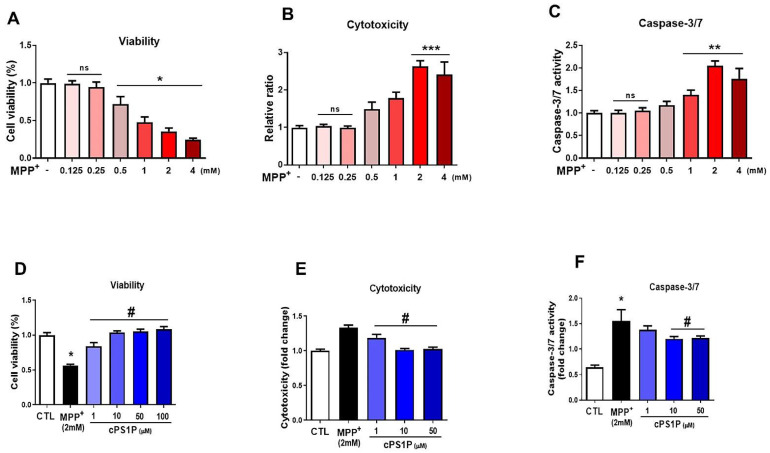
In vitro effects of cPS1P in the SH-SY5Y human neuroblastoma cells. (**A**–**C**) Cell viability, cytotoxicity, and caspase-3/7, respectively, profiles at different concentrations of MPP+ (n = 3). (**D**–**F**) Cell viability, cytotoxicity, and caspase-3/7, respectively, of MPP+ (2mM) and cPS1P (1, 10, 50 μM) treatment (n = 3). The values indicate the mean ± SEM. The significant differences have been given in the graphs. * Significant difference between the control and MPP+; # Significant difference between MPP+ and cPS1P treated. *p* < 0.05 was considered significant difference between the groups. * *p* < 0.05, ** *p* ≤ 0.01, *** *p* ≤ 0.001.

First, we performed in vitro assays using human neuroblastoma SH-SY5Y cells which are widely used in PD-related studies. The cytotoxicity profiles of MPP+ and cPS1P were examined via MTT assay. Six different concentrations (0.125, 0.25, 0.5, 1, 2 and 4 mM) of MPP+ were selected to measure cell viability, cytotoxicity, and caspase-3/7. The results obtained indicated that cell viability was decreased significantly at the doses of 0.5, 1, 2, and 4 mM in SHSY-5Y cells (Figure 1A), while increased significantly for cytotoxicity and neuronal apoptosis (Figure 1B,C). Next, the cells were exposed to MPP+ (2 mM) alone or co-treated with different concentrations (1, 10, and 50 µM) of the cPS1P to analyze cell viability, cell cytotoxicity, and neuronal apoptosis. The results revealed that SHSY-5Y cells treated with cPS1P significantly perpetuated the cell viability in a dose-dependent manner and significantly reduced the cytotoxic cell death and caspase3/7 activity (Figure 1D–F). 

### 3.2. cPS1P Alleviates Motor Dysfunctions Observed in MPTP and NSE-hαSyn PD Mouse Models

**Figure 2:**
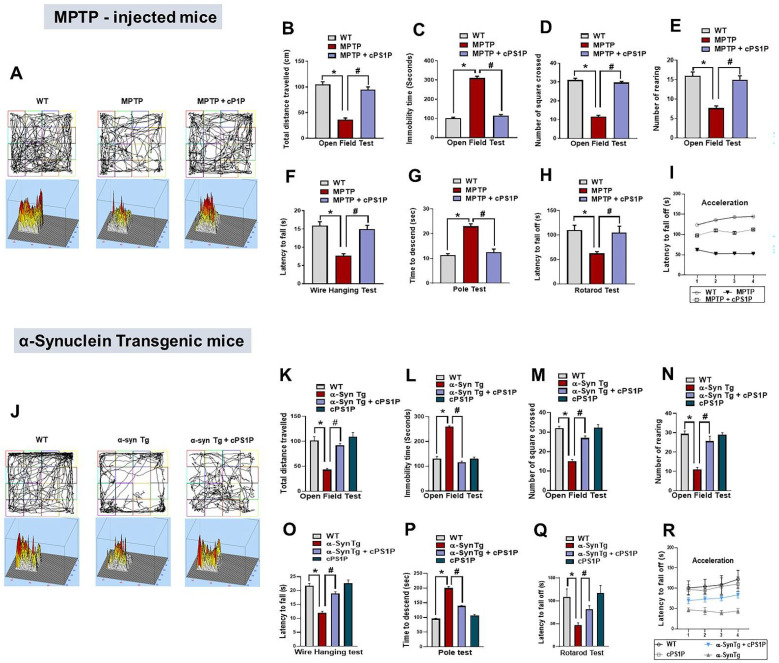
Effects of cPS1P against motor dysfunctions in the MPTP and NSE-hαSyn PD mouse models. (**A**,**B**,**J**,**K**) Graphical representation showing the total distance traveled by the mouse in the open field box of MPTP and NSE-hαSyn PD mouse models (n = 8 mice/group/model). (**C**,**L**) Graphical representation showing the immobility time of the mouse in the open filed box of MPTP and NSE-hαSyn PD mouse models (n = 8 mouse/group/model). (**D**,**M**) Graphical representation showing the number of squares crossed by the mouse in the open filed box of MPTP and NSE-hαSyn PD mouse models (n = 8 mice/group/model). (**E**,**N**) Graphical representation showing the mouse’s rearings in the open filed box of MPTP and NSE-hαSyn PD mouse models (n = 8 mice/group/model). (**F**,**O**) Graphical representation showing the latency to fall in the wire hanging test of MPTP and NSE-hαSyn PD mouse models (n = 8 mice/group/model). (**G**,**P**) Graphical representation showing the time to descend to the ground in the pole test of MPTP and NSE-hαSyn PD mouse models (n = 8 mice/group/model). (**H**,**I**,**Q**,**R**) Graphical representation showing the latency to fall off from the rotarod test of MPTP and NSE-hαSyn PD mouse models (n = 8 mice/group/model). The values indicate the mean ± SEM. The significant differences have been given in the graphs. * Significant difference between the control and model group; # Significant difference between the model and cPS1P co-treated mouse. *p* < 0.05 was considered significant difference between the groups, as measured with one-way ANOVA followed by Tukey’s multiple comparison test.

To analyze the effect of cPS1P on motor dysfunctions, we performed different behavior tests such as open field test, wire hanging test, pole test, and rotarod test. According to the open field test, the total distance traveled by the MPTP and NSE-hαSyn-induced PD mouse models were significantly reduced compared to the control group. Interestingly, this effect was markedly reversed in both PD model + cPS1P co-injected groups (Figure 2A,B,J,K). Second, in the open field test, the immobility time was significantly increased in the MPTP and NSE-hαSyn groups compared to the control group, but this effect was significantly reversed with the administration of cPS1P (Figure 2C,L). Next, the number of square crossings and rearing in the open field test showed significant increase in the cPS1P-treated group, compared to MPTP and NSE-hαSyn-induced models (Figure 2D,E,M,N). Furthermore, based on the wire hanging and a pole tests, cPS1P treated groups significantly preserved the hanging time and the descending time, respectively (Figure 2F,G,O,P). Lastly, the rotarod test, to assess the motor coordination and balance, showed that both PD models displayed shorter latency to fall off early than control group. However, cPS1P treatment significantly improved motor learning and showed longer latency to fall off than PD models (Figure 2H,I,Q,R). The overall behavioral findings suggested that cPS1P plays a role in restoring motor functions in both MPTP and NSE-hαSyn PD mouse models.

### 3.3. cPS1P Regulated the Expression of S1PR1 and α-Synuclein in the SNpc and Striatum of the MPTP and NSE-hαSyn-Induced PD Mouse Models

**Figure 3:**
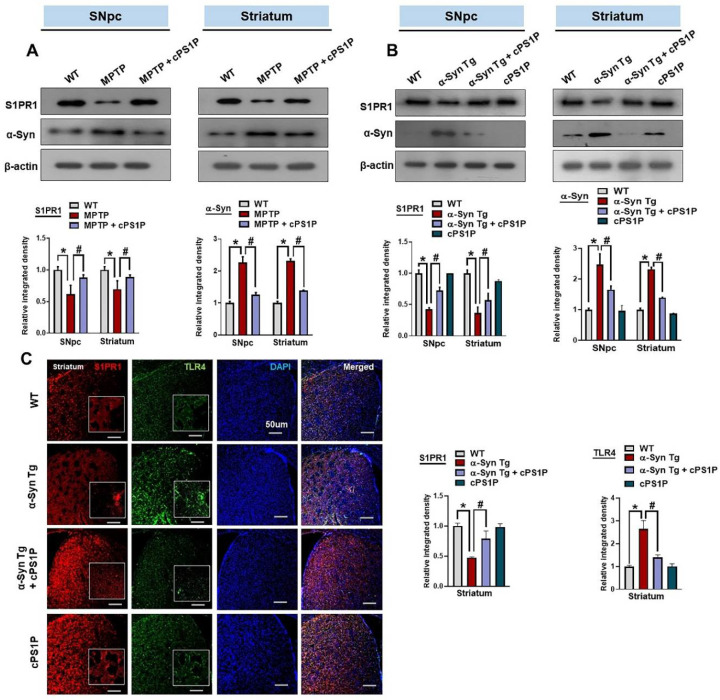
Effects of cPS1P against S1PR1 and α-synuclein in the MPTP and NSE-hαSyn PD mouse models. (**A**) Expression of S1PR1 and α-synuclein in the substantia nigra pars compacta (SNpc) and striatum of MPTP PD mouse model (n = 5 mice/group). (**B**) Expression of S1PR1 and α-synuclein in the SNpc and striatum of NSE-hαSyn PD mouse model (n = 5 mice/group). (**C**) Immunofluorescence analysis of S1PR1 and TLR4 in the striatum of NSE-hαSyn PD mouse model (n = 3 mice/group). The values indicate the mean ± SEM. The significant differences have been given in the graphs. * Significant difference between the control and model group; # Significant difference between the model and cPS1P co-treated mouse. *p* < 0.05 was considered significant difference between the groups, as measured with one-way ANOVA followed by Tukey’s multiple comparison test.

Through western blot analysis, we examined whether novel cPS1P is associated with S1PR1. The findings showed reduced expression of S1PR1 in the substantia nigra pars compacta (SNpc) and striatum of MPTP and NSE-hαSyn PD mouse models compared to the control group. This reduced expression was significantly restored in the cPS1P + MPTP/NSE-hαSyn PD mouse groups (Figure 3A,B). One of the underlying mechanisms of dopaminergic neurodegeneration is activating the innate immune system. Therefore, we checked the immunoreactivity of toll-like receptor 4 (TLR4). The results suggested an enhanced immunoreactivity of TLR4 with down-regulation of the S1PR1, as shown by the co-localization of S1PR1 and TLR4 (Figure 3C). We further explored whether the activation of the S1PR1 could reduce the main pathological hallmarks, α-Syn, in the brain of PD models. Our findings showed that the treatment of cPS1P to the PD mouse models significantly reduced the expression of α-Syn (Figure 3A,B).

### 3.4. Effects of cPS1P on the Expression of Dopamine-Related Proteins in the SNpc and Striatum of the MPTP and NSE-hαSyn PD Mouse Models

**Figure 4:**
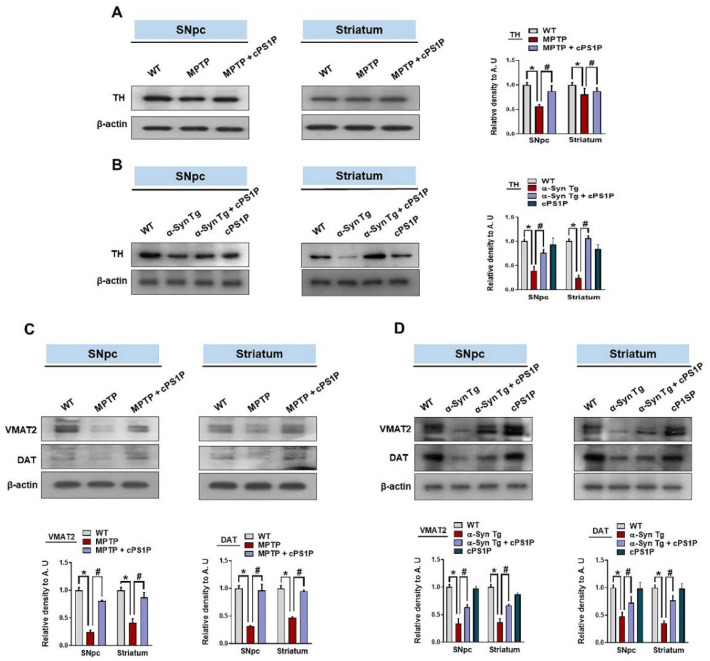
Effects of cPS1P on the expression of dopamine-related proteins in the MPTP and NSE-hαSyn PD mouse models. (**A**,**B**) Expression of TH in the substantia nigra pars compacta (SNpc) and striatum of MPTP and NSE-hαSyn PD mouse models, respectively (n = 5 mice/group/model). (**C**,**D**) Expression of VMAT2 and DAT in the SNpc and striatum of MPTP and NSE-hαSyn PD mouse models, respectively (n = 5 mice/group/model). The values indicate the mean ± SEM. The significant differences have been given in the graphs. * Significant difference between the control and model group; # Significant difference between the model and cPS1P co-treated mouse. *p* < 0.05 was considered significant difference between the groups, as measured with one-way ANOVA followed by Tukey’s multiple comparison test.

**Figure 5:**
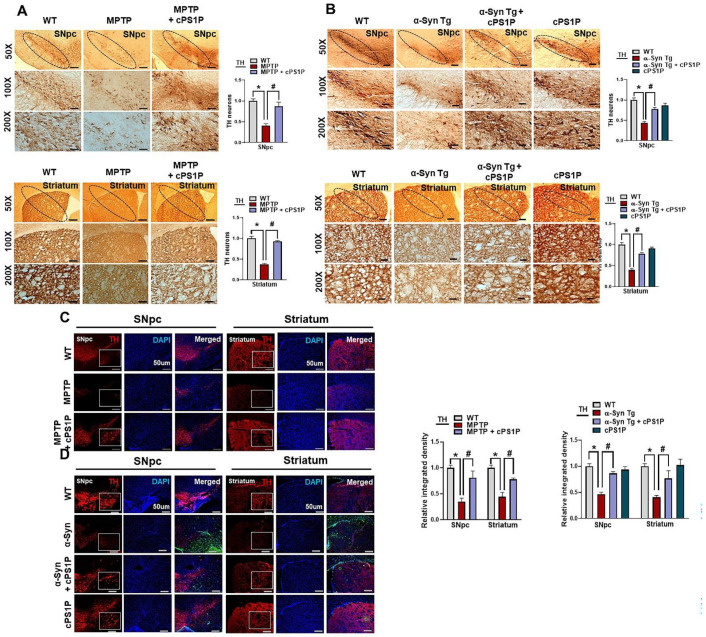
Immunostaining analysis of TH in the SNpc and striatum of MPTP and NSE-hαSyn PD mouse models. (**A**,**B**) Immunostaining of TH in the substantia nigra pars compacta (SNpc) and striatum of MPTP and NSE-hαSyn PD mouse models, respectively (n = 3 mice/group/model). (**C**,**D**) Immunofluorescence reactivity of TH in the SNpc and striatum of MPTP and NSE-hαSyn PD mouse models, respectively (n = 3 mice/group/model). The density values are relative to those of the control group and are expressed in arbitrary units (AU). Magnification 10x, scale bar = 50 µm. The values indicate the mean ± SEM. The significant differences have been given in the graphs. * Significant difference between the control and model group; # Significant difference between the model and cPS1P co-treated mouse. *p* < 0.05 was considered significant difference between the groups, as measured with one-way ANOVA followed by Tukey’s multiple comparison test.

Tyrosine hydroxylase (TH), vesicular monoamine transporter 2 (VMAT2), and dopamine transporter (DAT) are important for the synthesis and transport of dopamine. Based on our results, the expression of TH in the SNpc and striatum of the MPTP and NSE-hαSyn PD mouse models were significantly reduced compared to the control group, whereas the expression of TH was significantly upregulated with the administration of cPS1P (Figure 4A,B). In addition, our results also revealed low expression of VMAT2 and DAT in the SNpc and striatum in both PD models. On the contrary, cPS1P treatment significantly enhanced the expression of VMAT2 and DAT in the SNpc and striatum of both PD models (Figure 4C,D). Since TH is key regulator for the maintenance of dopaminergic neurons and in order to validate our western blot results, we evaluated the effects of cPS1P on TH expression in the brain of both PD models. According to our immunostaining results, the expression of TH was reduced in the SNpc and striatum in both PD models and administration of cPS1P significantly increased the level of TH (Figure 5A–C).

### 3.5. cPS1P Treatment Ameliorated Gliosis in the MPTP and NSE-hαSyn PD Mouse Brain

**Figure 6:**
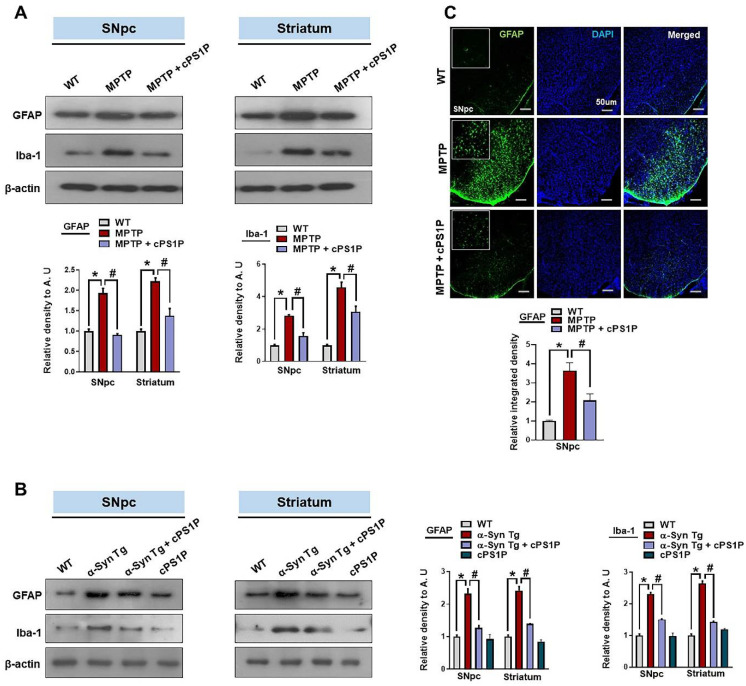
cPS1P treatment ameliorated PD-induced activation of glial cells. (**A**) Western blot analysis of GFAP and Iba-1 in the substantia nigra pars compacta (SNpc) and striatum of MPTP PD mouse model (n = 5 mice/group). (**B**) Western blot analysis of GFAP and Iba-1 in the SNpc and striatum of NSE-hαSyn PD mouse model (n = 5 mice/group). (**C**) Immunofluorescence reactivity of GFAP in the SNpc of MPTP PD mouse model (n = 3 mice/group). The density values are relative to those of the control group and are expressed in arbitrary units (AU). Magnification 10×, scale bar = 50 µm. The values indicate the mean ± SEM. The significant differences have been given in the graphs. * Significant difference between the control and model group; # Significant difference between the model and cPS1P co-treated mouse. *p* < 0.05 was considered significant difference between the groups, as measured with one-way ANOVA followed by Tukey’s multiple comparison test.

To unveil the underlying mechanisms responsible for the anti-inflammatory effects of cPS1P, we checked the expression of activated astrocyte and microglia via glial fibrillary acidic protein (GFAP) and ionized calcium-binding adaptor protein-1 (Iba-1) markers. Western blot results suggested that the expression of GFAP and Iba-1 were markedly upregulated in SNpc and striatum of MPTP and NSE-hαSyn mouse brain. Interestingly, these markers were significantly reduced in the cPS1P co-treatment (Figure 6A,B). Similarly, the immunofluorescence analysis also showed enhanced immunofluorescence of GFAP in the SNpc of MPTP-injected mouse brain, which was reduced in the cPS1P co-treated mouse brain (Figure 6C).

### 3.6. Effects of cPS1P on the Expression of Inflammatory Factors in the MPTP and NSE-hαSyn PD Mouse Models

**Figure 7:**
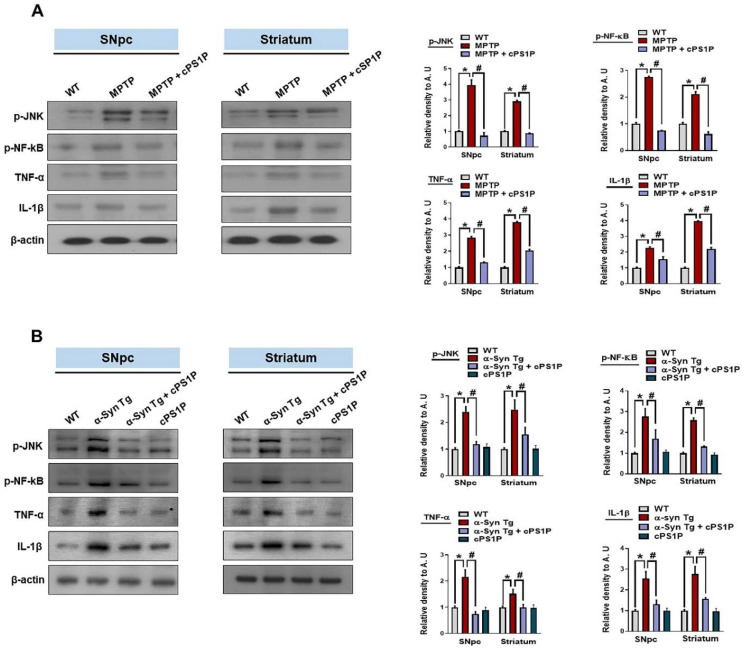
cPS1P prevents PD-induced activation of p-JNK and neuroinflammatory factors in the MPTP and NSE-hαSyn PD mouse models. (**A**,**B**) Western blot analysis of p-JNK, p-NF-κB, TNF-α, and IL-1β in the substantia nigra pars compacta (SNpc) and striatum of MPTP and NSE-hαSyn PD mouse models, respectively (n = 5 mice/group/model). The values indicate the mean ± SEM. The significant differences have been given in the graphs. * Significant difference between the control and model group; # Significant difference between the model and cPS1P co-treated mouse. *p* < 0.05 was considered significant difference between the groups, as measured with one-way ANOVA followed by Tukey’s multiple comparison test.

To elucidate the exact factors responsible for the neuroinflammation associated with PD and the effects of cPS1P on the inflammatory cytokines, we checked the expression of phosphorylated c-Jun N-terminal kinases (p-JNK), phosphorylated nuclear factor kB (p-NF-kB), tumor necrosis factor-alpha (TNF-α), and interleukin 1-beta (IL-1β) in the SNpc and striatum. The findings showed enhanced expression of all aforementioned markers in the MPTP and NSE-hαSyn PD mouse brain, compared to the control group. Interestingly, these markers were significantly reduced in the cPS1P co-treated mouse brain regions (Figure 7A,B).

### 3.7. cPS1P Treatment Reduced Neuronal Cell Death in MPTP and NSE-hαSyn PD Mouse Brain

**Figure 8:**
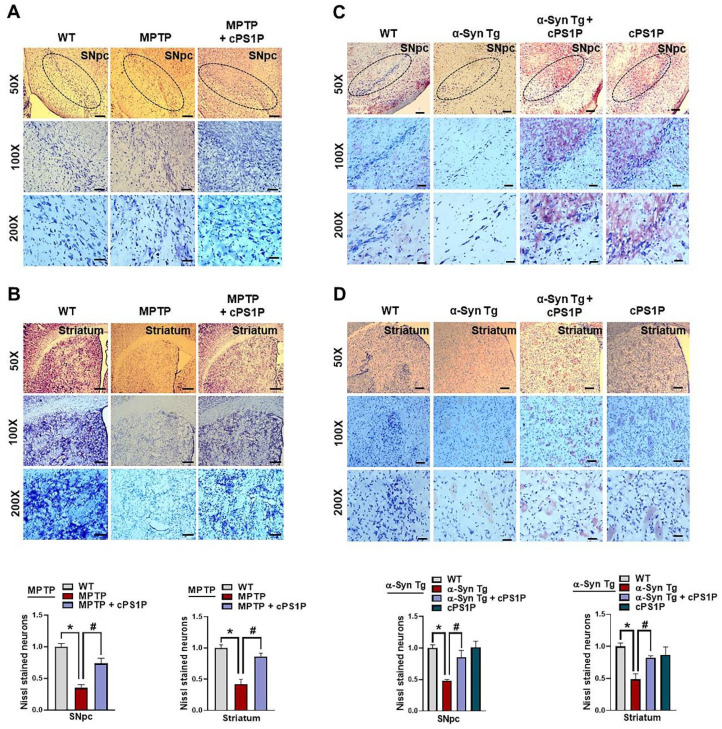
Effects of cPS1P against neuronal cell death in the SNpc and striatum of MPTP and NSE-hαSyn PD mouse models (**A**,**B**) Nissl staining of survival neurons in the substantia nigra pars compacta (SNpc) and striatum of the MPTP PD mouse model (n = 3 mice/group). (**C**,**D**) Nissl staining of survival neurons in the SNpc and striatum of the NSE-hαSyn PD mouse model (n = 3 mice/group). The values indicate the mean ± SEM. The significant differences have been given in the graphs. * Significant difference between the control and model group; # Significant difference between the model and cPS1P co-treated mouse. *p* < 0.05 was considered significant difference between the groups, as measured with one-way ANOVA followed by Tukey’s multiple comparison test.

To analyze the protective effect of cPS1P against MPTP and NSE-hαSyn-induced neuronal cell death, we performed Nissl staining on the brain sections. Both brain sections revealed reduced Nissl-stained neurons in the MPTP and NSE-hαSyn PD mouse brain, compared to the control group. Contrarily, this effect was significantly increased in both co-injected MPTP and NSE-hαSyn mouse brain (Figure 8A–D).

## 4. Discussion

In the present study, we investigated the effects of the novel S1P analog, cPS1P against neuropathological deficits associated with PD using MPTP and NSE-hαSyn PD mouse models. Our study showed that administration of cPS1P restored the motor dysfunctions associated with PD and cPS1P was associated with S1PR1 and α-Syn expression in the SNpc and striatum. Additionally, in both PD models, expression of dopamine-related proteins such as TH, VMAT2, and DAT were increased after cPS1P treatment in the SNpc and striatum. Furthermore, cPS1P treatment reduced inflammatory markers such as GFAP, Iba-1, p-JNK, p-NF-κB, TNF-α, and IL-1β in both brain regions. Therefore, the overall findings suggest that cPS1P has a protective property by reducing the neuropathological discrepancies associated with PD.

As dopamine plays a prominent role in normal physiological functions, dysfunction in the dopaminergic system is responsible for PD-associated symptoms [37]. MPTP- and αSyn-induced PD mouse models are widely used to study PD because it induces PD-like symptoms [38,39]. Furthermore, motor dysfunction is a prominent clinical feature of PD, and MPTP/α-synuclein-induced PD mouse have shown molecular alterations associated with Parkinson’s disease [38,39]. A recent study from Pepin et al. (2020), reported the neuroprotective properties of S1PR1 in MPTP-induced mouse model. The group administered S1PR1 selective agonist, SEW2871, and FTY720, which restored the motor abilities in the PD mouse model, a similar result as shown in our study [6]. TH is a rate-limiting enzyme in the brain for dopamine synthesis and is mainly used as a marker for dopaminergic depletion [40]. Both Pepin et al. and our data reported decrease in TH positive cells in SNpc, which was restored by treatments. Our study further showed that cPS1P also has the ability to provide neuroprotection in α-Syn-induced neurotoxicity. Furthermore, our results have shown decrease level of S1PR1 and increased level of α-Syn in the striatum and SNpc of MPTP and α-Syn-induced models, of which cPS1P treatment significantly restored both levels. Our findings suggest that the association between cPS1P with S1PR1 and α-Syn may be one contributing factor in rescuing dopaminergic neuronal loss and motor dysfunctions in PD.

As well as the disruption in the TH and related factors, neuroinflammation is also one of the hallmarks that play an essential role in the pathogenesis of PD. There are several stimuli for the initiation and progression of inflammation in the brain, and one of them is the sphingolipid metabolism alteration responsible for neuroinflammation. Studies have reported that alteration in sphingolipid metabolism causes glial cells activation and release of other inflammatory cytokines and mediators, which further cause neuronal cell death and neurodegeneration [3,41] and administration of FTY720 inhibits the mechanism of promoting neurodegeneration mediated by astrocytes and microglia [13]. Furthermore, activated glial cells are responsible for the release of p-JNK, p-NF-kB, which further modulate cytokine production and release proinflammatory cytokines such as TNF-α and IL-1β [42,43,44], which was in line with our study’s findings. Although this study did not measure the total JNK and NF-κB levels, in our group’s previous study, we have shown that the change in p-JNK and p-NF-κB was not due to the variation in total protein [45]. Moreover, previous studies showed that S1PRs regulation suppressed the expression of activated glial cells [13,46] and S1PR1 reduced cytokines in lipopolysaccharide (LPS) stimulated-mouse macrophages [47]. In line with these studies, our result shown that cPS1P treatment reduced activation of astrocyte, microglia, and inflammatory cytokines. In addition, the anti-inflammatory and anti-neurodegenerative effects of cPS1P was further shown in our Nissl staining, which showed that cPS1P significantly protected the dopaminergic neurons in the SNpc and striatum of both PD models. Similar to previous results, our results may be because cPS1P modulated the S1P receptor, thereby reducing inflammation-causing oxidative stress [19,25,48]. Studies have shown that S1PRs play a role in the regulation of lymphocytes trafficking and maintenance of vascular integrity [49,50]. Additionally, microglia play a prominent role in PD pathology and the balance between a pro-inflammatory or neurotrophic micro-environment [51]. Therefore, immunomodulatory drugs targeting S1PRs such as FTY720 or cPS1P may explain the reduced activation of immune cells and inflammatory cytokines. 

The main strength of this research was that we investigated the effect of cPS1P in two independent PD mouse models. Moreover, we have shown that the effect of cPS1P was in line with other S1PR modulators such as FTY720 and SEW2871. In addition, unlike those modulators, we have shown that cPS1P was associated with S1PR1 and the main PD pathology, α-Syn. However, there were some limitations in this study, such as using a mouse model to investigate PD. Rodent models are useful since they represent many aspects of PD symptoms, but animal models cannot replicate the exact pathophysiology of PD and often do not translate in clinical setting [52]. Nevertheless, mouse models are important tools in investigating PD and necessary before translating into the clinics.

## 5. Conclusions

Our findings support the notion that cPS1P shows a protective effect against PD pathology in MPTP- and NSE-hαSyn-induced mouse models. Therefore, future studies should investigate the mechanistic function of cPS1P in PD pathology.

## Figures and Tables

**Table 1:** List of antibodies.

Antibody	Host	Application	Manufacturer	Catalog number	Concentration
S1PR1	Rabbit	WB/IF	Invitrogen	PAI 1040	1:1000/1:100
α-Syn	Mouse	WB/IF	Santa Cruz Biotechnology	SC 58480	1:1000
TLR4	Mouse	IF	Santa Cruz Biotechnology	SC 293072	1:100
TH	Rabbit	WB/IF/IHC	Merck	AB152	1:1000/1:100/1:00
VMAT2	Rabbit	WB	Abcam	AB70808	1:1000
DAT	Rat	WB	Santa Cruz Biotechnology	SC 32259	1:1000
GFAP	Mouse	WB/IF	Santa Cruz Biotechnology	SC 33673	1:1000/1:100
Iba-1	Rabbit	WB	Thermo Fisher	PA527436	1:1000
p-JNK	Mouse	WB	Santa Cruz Biotechnology	SC 6254	1:1000
p-NF-κB	Mouse	WB	Santa Cruz Biotechnology	SC 136548	1:1000
TNF-α	Mouse	WB	Santa Cruz Biotechnology	SC 52746	1:1000
IL-1β	Mouse	WB	Santa Cruz Biotechnology	SC 32294	1:1000
β-actin	Mouse	WB	Santa Cruz Biotechnology	SC 47778	1:1000

## Data Availability

Data are contained within the article.
